# The use of Optical Character Recognition (OCR) in the digitisation of herbarium specimen labels

**DOI:** 10.3897/phytokeys.38.7168

**Published:** 2014-05-19

**Authors:** Robyn E. Drinkwater, Robert W. N. Cubey, Elspeth M. Haston

**Affiliations:** 1 Royal Botanic Garden Edinburgh, 20a Inverleith Row, Edinburgh, EH3 5LR, UK

**Keywords:** OCR, Digitisation, Data entry, Specimen, Label, Herbarium

## Abstract

At the Royal Botanic Garden Edinburgh (RBGE) the use of Optical Character Recognition (OCR) to aid the digitisation process has been investigated. This was tested using a herbarium specimen digitisation process with two stages of data entry. Records were initially batch-processed to add data extracted from the OCR text prior to being sorted based on Collector and/or Country. Using images of the specimens, a team of six digitisers then added data to the specimen records. To investigate whether the data from OCR aid the digitisation process, they completed a series of trials which compared the efficiency of data entry between sorted and unsorted batches of specimens. A survey was carried out to explore the opinion of the digitisation staff to the different sorting options. In total 7,200 specimens were processed.

When compared to an unsorted, random set of specimens, those which were sorted based on data added from the OCR were quicker to digitise. Of the methods tested here, the most successful in terms of efficiency used a protocol which required entering data into a limited set of fields and where the records were filtered by Collector and Country. The survey and subsequent discussions with the digitisation staff highlighted their preference for working with sorted specimens, in which label layout, locations and handwriting are likely to be similar, and so a familiarity with the Collector or Country is rapidly established.

## Introduction

There is an increasingly urgent need to document and make available the specimens held in herbaria and other natural history collections, particularly with the current biodiversity crisis (Berendsohn et al. 2010, Hardisty et al. 2013, Purves et al. 2013). The digitisation of the collections makes the data accessible for a wide range of taxonomic and ecological research being carried out around the world (e.g. Elith et al. 2006, Bebber et al. 2010, Lees et al. 2011, Lavoie 2013). The size of the collections held in major herbaria means that complete digitisation of the specimens they hold is often unfeasible, especially with the decreased funding at the present time.

At the Royal Botanical Garden, Edinburgh (RBGE), a large-scale project to digitise the collections has been running in which specimens are minimally databased (Haston et al. 2012a). Minimal data includes filing name and geographical region, as well as a barcode to act as a unique identifier. The high resolution, zoomable images of these specimens are made available through the online Herbarium Catalogue, accessed through the RBGE website (www.rbge.org.uk). They are also accessible via other online resources including Europeana (www.europeana.eu/) and Genbank (www.ncbi.nlm.nih.gov/genbank/) using a stable URI system (Hyam et al. 2012). Whilst additional label data are not initially captured, they can be accessed by examining the specimen online. There are approximately 3 million specimens in the herbarium at RBGE; of these 630,000 have been databased with 30% only having minimal data attached.

A similar approach is being used at the New York Botanic Garden Herbarium which holds an estimated 7.3 million specimens, where they have been databasing and imaging the collection for 17 years (Tulig et al. 2012). Based on the work already completed they recently estimated that it would take a further 600,000 hours to completely database and image the remaining approx. 6 million specimens. They have brought in new protocols for partially databasing specimens, increasing the speed of processing from an average of 10 per hour to 125 per hour.

Whilst further information can be found through looking at images, data that are useful for biodiversity studies and other research are not easily available, and cannot be extracted from the database for use. These data can include location, habitat and a description of the plant. The next step in the process of databasing specimens must be to find ways of creating more complete and useful records, whilst minimising the need for a large investment in staff hours.

It is only recently that Optical Character Recognition (OCR) has started to be used more widely to aid with the digitisation of natural history collections (Moen et al. 2008, Heidorn and Wei 2008, Nelson et al. 2012) and literature relating to these collections such as the Biodiversity Heritage Library (Biodiversity Heritage Library 2014) which uses OCR output to help navigate the literature. As the quality of the software has improved, OCR has become a viable option, more able to cope with the complex tasks which can be presented by natural history objects; e.g. distinguishing between labels and plant material on a herbarium specimen. Another contributing factor to the increased viability of OCR could be that there is now a large enough body of imaged specimens to make investment in OCR software worthwhile.

Several software applications have been developed to make use of OCR outputs easier. SALIX (Lafferty and Landrum 2008, Barber et al. 2013) and HERBIS (Beaman et al. 2006) parse the OCR output to a database, in a semi-automatic way, with the process being watched and facilitated by a user. Another approach (Heidorn and Wei 2008) has been to mark-up the output from the OCR, for input into the Darwin Core. Silver Biology (2013) is currently testing a site for enabling a citizen science initiative to database herbarium specimen labels. The OCR output is tagged with the relevant fields (e.g. Collector) and then parsed into Darwin Core fields. The use of OCR is also being explored by the AugmentOCR working group as part of Integrated Digitized Biocollections (iDigBio), the National Resource for Advancing Digitization of Biodiversity Collections (ADBC) funded by the National Science Foundation.

At RBGE we have been exploring how OCR processing can be used to add data to the minimal entries already created for specimens.

Whilst we have only just started to make use of data from OCR, the process of gathering this information has been integrated into the digitisation workflows since 2010. The workflows at RBGE have been developed in such a way that they are ‘modular’ (Haston et al. 2012b), to allow flexibility in the stages of digitising specimens. All specimen images are passed through ABBYY Recognition Server (Abbyy 2014) which provides the OCR output in the form of a text file. The unparsed text is automatically entered into a single field within a MySQL database. A PDF file with the OCR output overlaid on the image of the specimen is also saved.

The aims of this investigation are to examine how we can incorporate the OCR output into the workflows to make the digitisation process more efficient.

In particular we hope to be able to answer the following questions:

1. Can OCR speed up the digitisation process, whilst maintaining data quality?

2. Is OCR worth the investment in time and software?

## Methods

To investigate how data extracted from OCR process can aid in the addition of data to minimal database records, a series of trials were carried out by six members of the digitisation team at RBGE.

**Figure 1.**
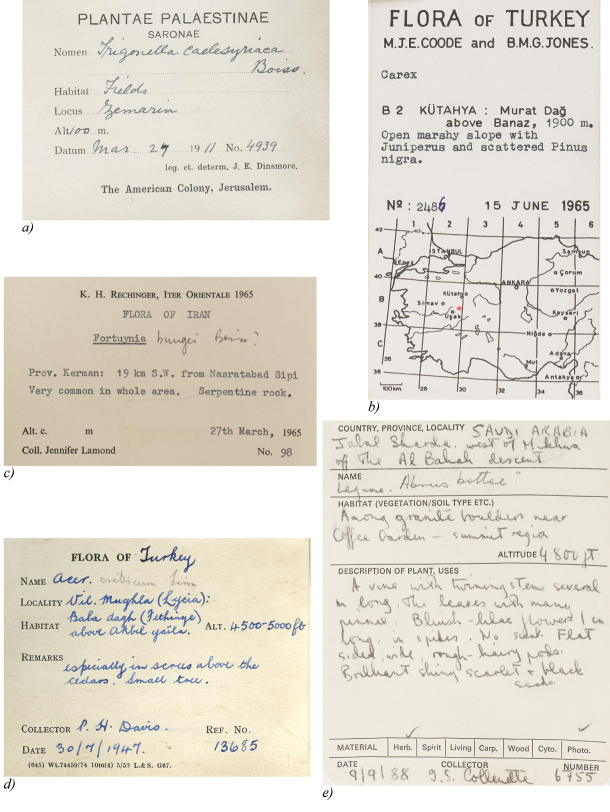
Example labels: **a** Pre-printed label with handwritten details **b** and **c** mixed labels with pre-printed and typed information **d** Mainly handwritten label, with printers mark **e** Mainly handwritten label with unusual phrasing.

The specimens used in this study were collected in Southwest Asia and the Middle East from the early 19^th^ century to the present day. The earlier specimens are generally handwritten, but some have printed headings (Figure 1a). Later specimens are generally type-written or printed (Figure 1b and 1c). Specimens include those used in the writing of the Flora of Turkey (Davis 1985), and also the ongoing work on the Flora of Arabia (Miller 1996). This is a key focus region for research at RBGE and there are several members of staff who have considerable experience of collections from this area and so they offer a valuable resource, being able to offer advice on difficult handwriting, cryptic notes on labels and terms to use when searching OCR text.

These specimens have been imaged and minimally databased as part of an ongoing project to image and digitise RBGE herbarium specimens. The digitisation workflow includes the routine processing of all specimen images through ABBYY Recognition Server software, and the unparsed text output is stored within the images database.

For this study 20,000 specimen records were exported from the main database into a temporary Access database. The data included the minimal data fields, the image file location and the OCR data. The OCR output was searched for Countries and Collector names, which were considered to be the most useful additional fields, as well as being the most likely to be easily ‘read’ by the OCR software. A short SQL script in Access was used to search for a selected word within the OCR text and, when present, to copy the word to a new field. As the specimens were from a limited geographical area, it allowed a list of Countries and major Collectors to be developed.

As well as carrying out simple searches for Country and Collector, other keywords and phrases were found to be peculiar to a particular Collector or Country. These included printers marks (Figure 1d) on otherwise handwritten labels, unusual wording (Figure 1e) or abbreviations used within pre-printed label headings. Common ‘reading’ errors made by the OCR software (e.g. the OCR software reading Turbey instead of Turkey), variations in spellings of provinces, states or cities were also useful in attaching an initial Country or Collector to a specimen.

The specimen records were then sorted by either Country or Collector, to enable verification of the data. This could be done rapidly using IrfanView (2014) a freeware graphic viewer, which was able to use the image file locations to create a slideshow of specimen images. This allowed specimens to be rapidly checked and the Collector and/or Country to be verified.

Once the Collector and Country had been verified, these data were added to the original specimen records using a batch process facility. This allowed a large number of records to be rapidly updated.

### Trial format

The updated records were then used as the basis of a series of trials to assess how the data extracted from the OCR could be utilised in the wider digitisation effort at RBGE. The trials were set-up to look at rates for data entry with and without OCR data being used to aid the process.

The digitisation staff used the institutional database for data entry, allowing full use of the look up tables for collectors, countries and their top-level divisions, as well as a short-cut for repeat entry of fields. They were provided with two screens, one landscape and one portrait to allow for easy viewing of specimen images and organisation of other programmes required.

Each trial consisted of two protocols. These protocols differed in the amount of data being captured. The Complete Protocol involved the capture of all data on the specimen, including the original label as well as any additional determinations and annotations. The Partial Protocol limited the capture of data to a pre-determined standard set of fields including collector, collection number and date, locality information, and the taxon name under which it was originally collected. Twenty-four batches of records, each comprising 50 specimens, were created using a series of filters. These batches were then given to the team of digitisers.

**The six ‘filters’ used were:**

1. Pre-study control (Random)

2. Collector only

3. Country only

4. Collector and Country

5. Collector and Country, with full OCR output

6. Post-study control (Random)

Trial 1: Pre-study control

This first trial was used as a control and provided a baseline for the testing. The digitisers were each given two batches of randomly selected specimens which only contained minimal data.

Trial 2: Collector only

The digitisers were each given two batches of specimens which had been selected using a filter which ensured that all specimens in the batch had been collected by the same collector or collector group.

Trial 3: Country only

The digitisers were each given two batches of specimens which had been selected using a filter which ensured that all specimens in the batch had been collected in the same country.

Trial 4: Collector and Country

The digitisers were each given two batches of specimens which had been selected using a filter which ensured that all specimens in the batch had been collected in the same country and by the same collector or collector group.

Trial 5: Collector and Country, with full OCR output

The digitisers were each given two batches of specimens which had been selected using a filter which ensured that all specimens in the batch had been collected in the same country and by the same collector or collector group. In addition, a full OCR output was also provided. For this study the type of OCR output used was one where a PDF of the OCR output, layered over the top of the specimen image where text was detected. The digitisers were then asked to copy the OCR data into the appropriate fields and correct it for spelling and punctuation errors.

Trial 6: Post-study control

This final trial was used as a second control to assess how using the other methods, and increased familiarity with the process affected timings. The digitisers were each given two batches of randomly selected specimens which only contained minimal data.

The digitisers were asked to keep a record of the time it took to complete each set of specimens, excluding breaks.

### Analysis

The results of the tests were collated and an Analysis of Variance (ANOVA) was carried out in R. The digitisers were also asked to complete a short survey which explored the ‘people’ side of the work, asking about preferred workflows, their perception of time taken to complete tests and what resources may be of benefit to aid similar work in the future. The online questionnaire was followed up with an informal discussion of the trials, allowing points mentioned in the survey to be discussed further and also to discuss some of the wider implications of digitising specimens.

## Results

The results of the study show significant differences in the average time taken for the trials to be completed. The level of variation observed between the trials differed between the Complete and Partial Protocols. Significant variation was observed between the trials completed using the Partial Protocol.

The results are summarised in Table 2.

**Table 1.** Format of the trials.

Trial	‘Filter’	Protocol	Number of repeats/person	Total specimens/person
1.	Random	Complete	2	100
		Partial		100
2.	Collector	Complete	2	100
		Partial		100
3.	Country	Complete	2	100
		Partial		100
4.	Collector & Country	Complete	2	100
		Partial		100
5.	Collector & Country(OCR)	Complete	2	100
		Partial		100
6.	Random	Complete	2	100
		Partial		100

**Table 2.** Average time taken to complete trials.

Trial	‘Filter’	Number of completed batches per Protocol	Average Complete Protocol (minutes)	% time saved (compared with Random 1)	Average Partial Protocol (minutes)	% time saved (compared with Random 1)
1.	Random 1	10	313	0%	226.9	0%
2.	Collector	10	259.5	17.1%	220.2	2.7%
3.	Country	10	345.7	10.5% increase	192.6	15.2%
4.	Collector & Country	10	262.8	16.1%	105.3	53.6%
5.	Collector & Country (OCR)	10	252.6	19.3%	125.7	44.7%
6.	Random 2	10	283.9	9.3%	219.9	3.1%

Diagnostic plots were used to check that the data were normally distributed. There was evidence for some heteroscedascity in the data, so we cannot assume a normal distribution. A Poisson distribution was tested and compared with a normal distribution using AIC, which suggested that a normal distribution model fits the data better than a Poisson distribution model. We therefore present the results from the analyses based on a normal distribution.

The data were analysed to investigate the effect of Person on the trials, since this would have an impact on the analysis used. Firstly a linear regression was carried out treating each person as a factor. This suggested that the variation observed is explained by the Trial rather than the Person. Secondly co-plots were used to visualise the interactions of the person and the trials. These showed no significant effect of the person on the results, and the major effects were related to Trial. As a result of these analyses it was decided that one of the datasets should be excluded from the analyses as an outlier.

The Analysis of Variance (ANOVA) showed significant variation between the 12 trials.

**Figure 2.**
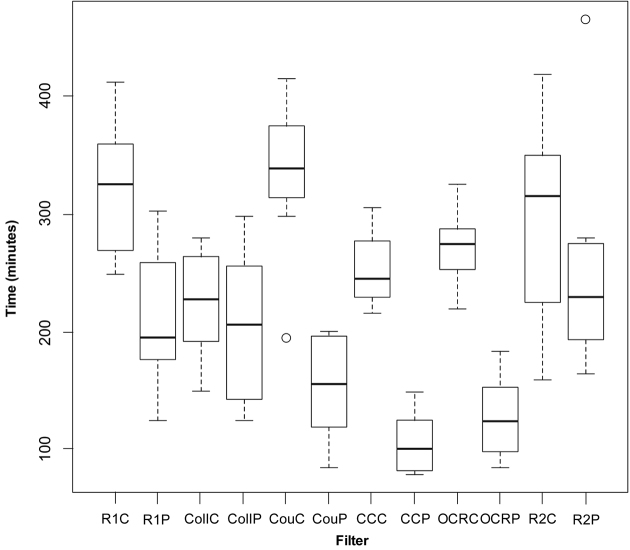
Box plot of Complete and Partial Protocol results. R1C – Random 1 complete; R1P – Random 1 Partial; CollC – Collector only Complete; CollP – Collector only Partial; CouC – Country only Complete; CouP – Country only Partial; CCC – Collector & Country Complete; CCP – Collector & Country Partial; OCRC – Collector & Country OCR Complete; OCRP – Collector & Country OCR Partial; R2C – Random 2 Complete; R2P – Random 2 Partial.

**Figure 3.**
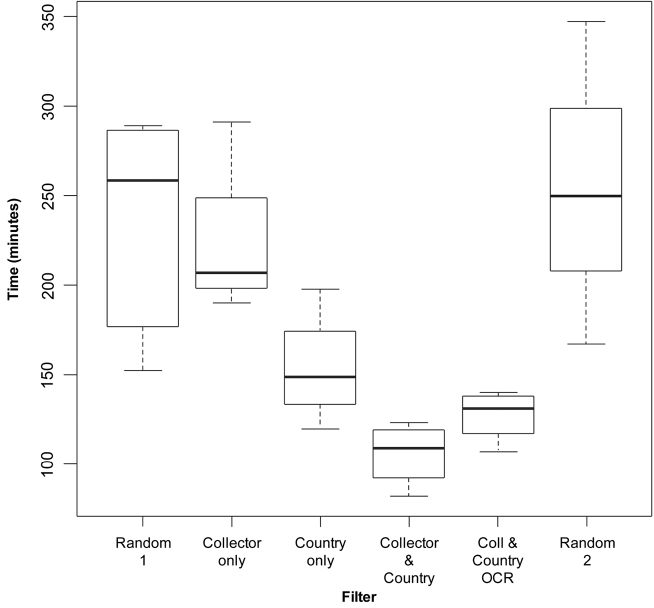
Box plot of Partial Protocol results.

The filters appear to have greater impact in the trials using the Partial Protocol. The Partial Protocol is used as the standard for the majority of databasing at RBGE. Therefore these trials were analysed further to explore this impact and the results are illustrated in the box plots (Figures 2 and 3).

### Partial protocol

The trials completed using the Partial Protocol show a significant reduction in the average time taken to add data to specimens which had been filtered by Country, by Collector and Country and by Collector and Country with OCR.

The greatest reduction in average times was seen in those specimens filtered by Collector and Country. The Country filter appeared to have the greatest impact on reducing the time.

The results of the ANOVA for the 6 trials are shown in Table 4. These were calculated using the Protocol ‘pairs’ (Complete and Partial). Three of the trials were found to have a result which was significant to greater than 0.001.

**Table 3.** Result of ANOVA for the 12 trials.

	Df	F Value	Pr (>F)	Significance
Trial	11	13.03	4.11e-14	*** (0)
Residuals	85			

**Table 4.** Result of ANOVA using Protocol ‘pairs’ (Complete and Partial).

Trial		Df	F Value	Pr (>F)	Significance
Partial	Trial	5	6.487	0.0013	** (0.001)
	Residuals	18			

### Survey

The survey was completed by all those who took part in the trials. The first five questions asked the digitisers to assign a value of 1-5 to each of tests, based on speed, ease of use, accuracy and preference.

Question 1: speed

The participants perceived the trials filtered by Country and Collector to be the fastest (66.7%) and the two random trials to be the slowest (66.7%).The use of OCR data to filter the specimens was perceived to be slightly faster than the Country only filter.

Question 2: ease of use

A similar result was found for the question asking the participants to rate the filters by their relative ease of use. Collector and Country was perceived to be the easiest to use (100%) and the hardest were the two random filters (66.7%).

Question 3: accuracy

Again the Collector and Country filter was perceived to be the least likely to lead to mistakes (83.3%) and the random filters were perceived to be the most likely to lead to mistakes (66.7% and 50%).

**Figure 4.**
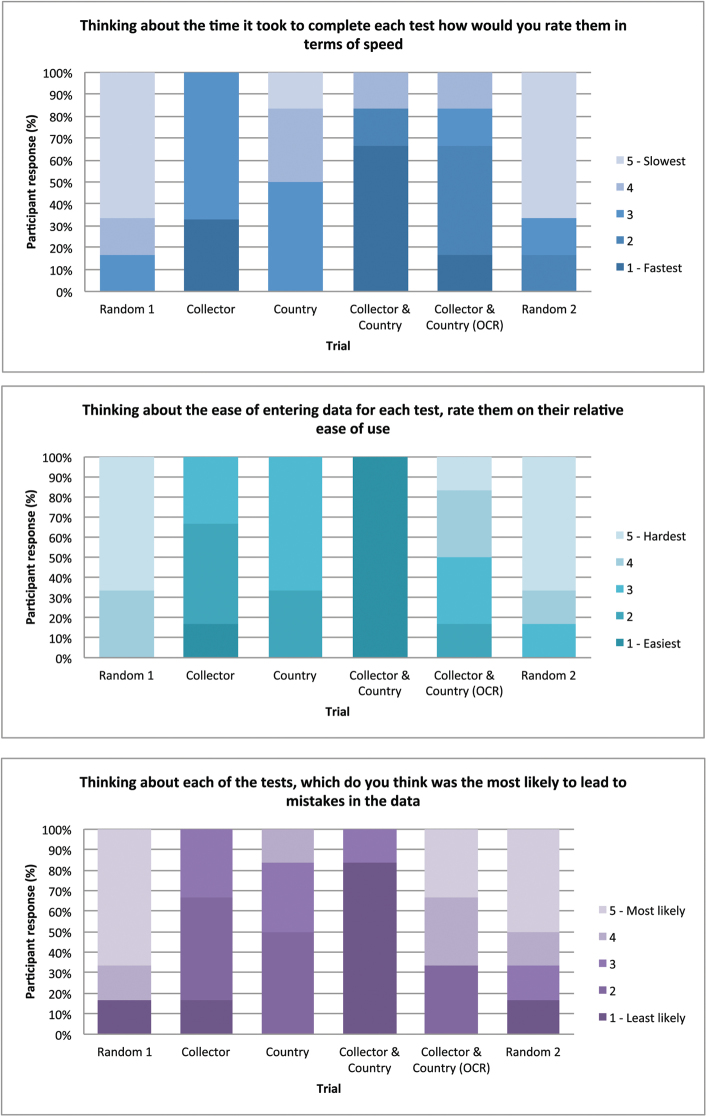
Digitiser responses to questions 1, 2 and 3 of survey

Questions 4 and 5: preference

The digitisers were asked which of the workflows would be preferred for digitising 50 and 1000 specimens. For 50 specimens there was a clear preference for the Collector and Country filter, with all participants selecting this filter. However when considering larger numbers of specimens the number selecting Collector and Country dropped, with 2 selecting the Country only filter.

## Discussion

### Summary

This study investigated how data extracted from OCR can be used to sort specimens prior to databasing and aid in the addition of data to minimal database records. Of the methods tested here, the most successful in terms of efficiency used the Partial Protocol, filtered by Collector and Country. This method was on average 20 minutes (8.9%) faster per batch of 50 records than the next most efficient method.

### Protocols: Complete and Partial

As expected, the Complete Protocol which requires a larger quantity of data to be entered for each record resulted in a significant increase in the time taken to enter data. In particular, the need to enter multiple specimen determinations may often involve the creation of additional name records not already held in the database which can be time consuming. The amount of data on a label to be entered is a balance between usefulness and cost. For most users, we believe that the Partial Protocol, which places more emphasis on the geographical data, captures the highest priority information from the label.

### Filters: Collector and Country

Prior to the trials, there had been an expectation that filtering the records by Collector would have the greatest impact. This was not borne out during the trials. In fact, the greatest impact came from filtering the records by Country. From the feedback it was apparent that a familiarity with the geography of a country aids the digitisation process more than familiarity with a Collectors label style and handwriting. However, a combination of the Country filter with the Collector filter was found to be most effective in speeding up the data entry process.

This was also reflected in the feedback from the digitisation team, who all identified this combined filter as the preferred option for digitising 50 specimens, and the majority would prefer this filter when digitising 1000 specimens. However, occasionally working with a large batch of similar records from a particular collector or country which were difficult in terms of legibility or geography resulted in reduced job satisfaction.

### Variability

Whilst some of the trials showed a much greater variation in times to complete than others, the lack of variation between the preliminary random trial and the final random trial suggests that there was little ‘learning effect’.

### Direct use of OCR data

The direct use of OCR output seems to have had very little effect on the time it took to digitise images. This may be due in part to the format of the output which did not allow users to copy multiple lines of text easily. More suitable output formats may increase the impact of the OCR output in the future.

The OCR output was most useful for long sections of text, often descriptions of the habitat and plant. However, some of the digitisers also found the output useful for shorter sections of texts, particularly place names.

In general, care needs to be taken in using the OCR output directly, as there can be some errors in punctuation, spelling and spacing. It is currently only of use for typed and printed labels, and not yet able to pick up hand-written ones, and so wasn’t available for all specimens encountered. In some cases the quality of the OCR output was so poor (spelling errors etc.) that it was quicker to type even the longer sections of text.

### The Human factor

The results of the questionnaire and the subsequent discussion with the digitisers resulted in several interesting and unexpected points.

### Preference for working with physical specimens

There was a clear preference expressed for working with physical specimens. One interesting point which was raised during the discussion with the digitisers, and which the authors hadn’t previously considered, was the preference for working with the actual specimen as opposed to the image of the specimen. Two main reasons for this came out of the discussion. Firstly they found that using a screen to view, read and interpret the label information can cause more strain on the eyes than looking at a physical specimen. Secondly they felt that the images of the specimens took more time to manipulate and access the label information. The software we had provided the digitisers did not allow an easy zoom to the area of interest, whereas they felt that a physical specimen can be manipulated more easily and moved to make the label easier to read.

### Working ‘methods’

The digitisers also expressed the view that it was desirable for two people to work on similar sets of specimens since this gave them the opportunity to discuss and help each other. This was something which was not designed as part of these trials, but which came about because of the selection of specimen sets. This was more apparent for one set of specimens in which the handwriting on the labels was particularly difficult to read.

For the purpose of the trials we pre-filled some of the fields in the institutional database: Collector, Country or both, depending on the trial. The work carried out in the preparation of the batches which allowed the pre-filling of these fields meant that some issues, such as difficult handwriting of a collector’s name, did not have to be handled by the digitisers. This was seen as an advantage by the digitisation team.

In the questionnaire we asked the digitisation team to complete, we asked whether they thought any filters would lead to an increase, or reduction in mistakes in the data. Whilst this is something we haven’t quantified by checking the data entered during this investigation, it is interesting to note that the Collector and Country filter was felt to be least likely to lead to mistakes in the data.

### Future work

This feedback from the digitisers has influenced how the next phase of the digitisation of the collection will develop. Where appropriate the digitisers will work in pairs enabling sharing of learning and expertise, and allowing discussion of problems encountered. Further to this, the digitisers felt it would be beneficial to have a resource which provided examples of collector’s hand-writing and locations for old or difficult names. There is also a need to take in to consideration the well-being of the digitisation staff, particularly with reference to the physical environment for repetitive tasks, something we will consider when developing the digitisation process in the future.

The use of OCR data will continue to be expanded for the digitisation of the collections in general. In particular this output is also likely to be of high quality for many of the more recent specimens, as they have clear type-written labels. For families like the Zingiberaceae where the labels often have very long descriptions, partly because floral characters are lost once the specimen is pressed, access to the OCR output of the label would allow the full label to be easily added to the specimen record through a simple cut and paste. A future study of how working with physical versus virtual specimens and how this affects work flows for the digitisation process may be carried out in the future to help optimise practices at RBGE.

We are exploring other elements we could extract from the OCR output. These include numerical elements such as the Collection Number, Date, Latitude and Longitude, and Altitude. There is also potential to extract additional levels of locality information.

Some of the processes for pre-sorting herbarium specimens described here may be used in the future as part of crowd-sourcing projects. Opening up the data entry process beyond the trained digitisation staff would require the implementation of quality control checks which have not been carried out in this study.

Whilst we have found that the quality of OCR output to be variable depending on the condition of the label, it is expected that the software will continue to improve, allowing increasing amounts of data to be extracted.
